# Susceptibility of Pancreatic Beta Cells to Fatty Acids Is Regulated by LXR/PPARα-Dependent Stearoyl-Coenzyme A Desaturase

**DOI:** 10.1371/journal.pone.0007266

**Published:** 2009-09-29

**Authors:** Karine H. Hellemans, Jean-Claude Hannaert, Bart Denys, Knut R. Steffensen, Cindy Raemdonck, Geert A. Martens, Paul P. Van Veldhoven, Jan-Åke Gustafsson, Daniel Pipeleers

**Affiliations:** 1 Diabetes Research Center, Brussels Free University-VUB, and JDRF Center for Beta Cell Therapy in Diabetes, Brussels, Belgium; 2 Department of Biosciences and Nutrition, Karolinska Institutet, Novum, Stockholm, Sweden; 3 LIPIT, Departement Moleculaire Celbiologie, Katholieke Universiteit Leuven-KUL, Leuven, Belgium; 4 Center for Nuclear Receptors and Cell Signaling, Department of Cell Biology and Biochemistry, University of Houston, Houston, Texas, United States of America; University of Bremen, Germany

## Abstract

Chronically elevated levels of fatty acids-FA can cause beta cell death in vitro. Beta cells vary in their individual susceptibility to FA-toxicity. Rat beta cells were previously shown to better resist FA-toxicity in conditions that increased triglyceride formation or mitochondrial and peroxisomal FA-oxidation, possibly reducing cytoplasmic levels of toxic FA-moieties. We now show that stearoyl-CoA desaturase-SCD is involved in this cytoprotective mechanism through its ability to transfer saturated FA into monounsaturated FA that are incorporated in lipids. In purified beta cells, SCD expression was induced by LXR- and PPARα-agonists, which were found to protect rat, mouse and human beta cells against palmitate toxicity. When their SCD was inhibited or silenced, the agonist-induced protection was also suppressed. A correlation between beta cell-SCD expression and susceptibility to palmitate was also found in beta cell preparations isolated from different rodent models. In mice with LXR-deletion (LXRβ^-/-^ and LXRαβ^-/-^), beta cells presented a reduced SCD-expression as well as an increased susceptibility to palmitate-toxicity, which could not be counteracted by LXR or PPARα agonists. In Zucker fatty rats and in rats treated with the LXR-agonist TO1317, beta cells show an increased SCD-expression and lower palmitate-toxicity. In the normal rat beta cell population, the subpopulation with lower metabolic responsiveness to glucose exhibits a lower SCD1 expression and a higher susceptibility to palmitate toxicity. These data demonstrate that the beta cell susceptibility to saturated fatty acids can be reduced by stearoyl-coA desaturase, which upon stimulation by LXR and PPARα agonists favors their desaturation and subsequent incorporation in neutral lipids.

## Introduction

Chronically elevated levels of saturated fatty acids may cause a reduction in beta cell mass during the pathogenesis of type 2 diabetes. Supporting evidence comes primarily from animal models and in vitro studies in which high fatty acid concentrations induce beta cell dysfunction and death [1]-[4]. Depending on the laboratory model, the lipotoxic process is seen (in)dependently of glucotoxic influences [5]. In cultures of purified rat beta cells, we found that palmitate was cytotoxic irrespective of the glucose concentration but that the cells varied in their susceptibility, some of them surviving while others rapidly or progressively proceeded to necrosis or apoptosis [6], [7]. This observation is another illustration of the functional heterogeneity in the beta cell population whereby cells differ in their individual sensitivities and/or defense mechanisms [8]-[10]. Fatty acid (FA) toxicity was found inversely related to the cellular ability to incorporate them as neutral lipids in the cytoplasm [6]. The capacity to form cytoplasmatic lipid droplets may thus serve as a cytoprotective mechanism by preventing accumulation of toxic non-esterified FA [6], [11]. Unsaturated FA such as oleate can channel palmitate into triglyceride pools away from pathways leading to cellular apoptosis [12]-[14]. A second protective mechanism consists in increasing breakdown of fatty acids in the beta cells [15], [16]. We recently demonstrated that the cytoprotective effect of PPARα agonists against long-chain fatty acid toxicity is associated with increased rates of β-oxidation and peroxisomal activity [7]. During the latter study we noticed that PPARα agonists also induced a higher expression of stearoyl CoA Desaturase (SCD), which is known to generate monounsaturated FA from palmitate and thus facilitates their incorporation into lipid reserves [17], [18], instead of their accumulation as non-esterified fatty acids or via an increased incorporation in ceramides [19]. Elevated SCD-levels have been shown to protect against saturated fatty acids in a number of cell types, including MIN6 cells [19]-[21]. The present study was undertaken to investigate the potential contribution of SCD in the detoxifying action of PPARα agonists.

Since the SCD1 and 2 enzymes are direct downstream targets of Liver X Receptors (LXR) [22], we started by examining whether LXR agonists can protect beta cells against palmitate through an SCD-dependent mechanism. LXRα and LXRβ are nuclear oxysterol receptors with established roles in cholesterol, lipid, and carbohydrate metabolism [23], [24], and have recently been shown to play an important role in maintaining beta cell function [25], [26].

## Results

### Activation of LXR protects beta cells against palmitate toxicity

**Figure 1:**
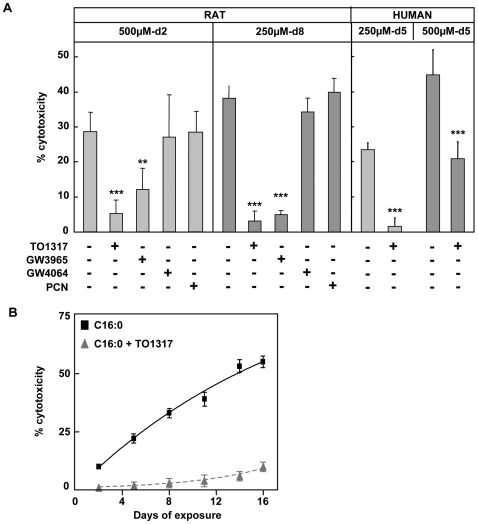
Agonists for LXR protect primary beta cells against palmitate toxicity. a) Rat and human beta cells were exposed to 500 µM-P, or 250 µM-P in the absence or presence of agonists for LXR (10 µM TO1317 or 1 µM GW3965), FXR (5 µM GW4064), or PXR (30 µM PCN). Data shown as mean+sd, n = 4-10, * p<0.05, ** p<0.01, *** p<0.001, versus palmitate b) Time course over 16 days for the toxicity of 250 µM-P±TO1317 (1 µM) in rat beta cells. Data shown as mean+sd, n = 7-10.

Rat beta cells were cultured with palmitate (C16:0) in presence or absence of LXR agonists. Culture with 500 µM palmitate-2 days (500 µM-P) or 250 µM-8 days (250 µM-P) resulted in a cytotoxicity of 29 and 38% respectively (Figure 1a). The LXR agonists TO1317 and GW3965 protected against both conditions keeping cell death below 10%. On the other hand, no protection was seen with the FXR and PXR agonists GW4064 and pregnenolone carbonitrile (PCN), which supports LXR specific protection. TO1317 was also found to protect human beta cells (Figure 1a). The protective effect of TO1317 was maintained over 16 days: 10±2% dead cells at 250 µM-P with the agonist versus 55±5% without (Figure 1b). Protection was also observed when TO1317 was added 24 h after starting cultures with 250 µM-P: 16±8% dead cells after 8 days versus 40±5% in its absence (P<0.01) (data not shown). None of the tested agonists influenced beta cell survival in absence of palmitate.

Pre-treatment of beta cells with TO1317 also protected against a subsequent exposure to 250 µM-P (Table 1). During a first period of 8 days, beta cells were cultured with or without the agonist; 250 µM-P was added on day 8 and culture continued without agonist. Protection was sustained up to 8 days in cells exposed to palmitate after removal of the protective agonist: 23±3% dead cells versus 38±3% (P<0.05). Beta cells cultured under control conditions for 8 days showed a comparable cytotoxic response as freshly isolated cells when subsequently exposed to palmitate for 2 to 8 days, indicating that prolonged culture did not alter their susceptibility for palmitate.

**Table 1:** Effect of TO1317 pretreatment on palmitate toxicity.

		*Percent cytotoxicity*	
**Pre-culture**	**Final condition**	**2 days**	**8 days**
Control	C16:0	14±2	38±3
TO1317	C16:0+TO1317	**1±2** $	**12±3** $
TO1317	C16:0	**5±3** $	**23±3** $
C16:0+TO1317	C16:0	**4±2** $	32±6

Beta cells were pre-cultured for 8 days in absence or presence of 1 µM TO1317 or 250 µM-P; on day 8 these conditions were replaced by the final conditions, as indicated, and the resulting cytotoxicities were measured after 2 or 8 days. Mean±SD, n = 4.

$ p<0.001, versus control.

In contrast to PPARα agonists that induce protection to saturated and mono-unsaturated FA [7], TO1317 did not protect against palmitoleate (C16:1), vaccenate (C18:1 n-7) or oleate (C18:1 n-9), while it protected against stearate (C18:0) (Table S1).

### Effect of LXR agonist on intracellular lipid accumulation

**Figure 2:**
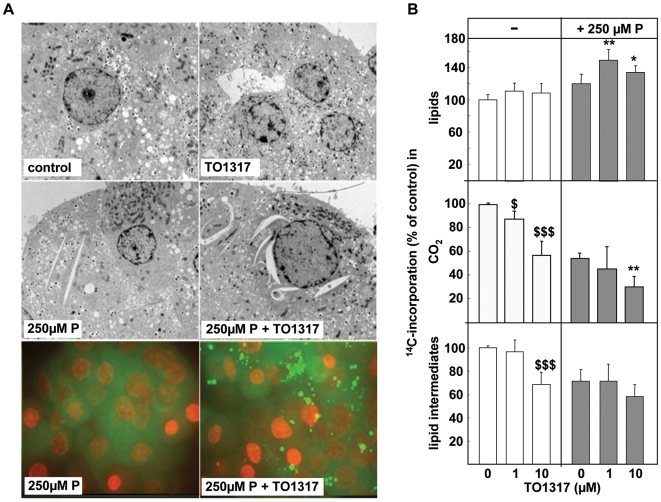
Effect of LXR agonist on intracellular lipid accumulation and palmitate metabolism. a) Representative figure showing electron microscopy and vital nile red staining on control cells, and beta cells exposed to 250 µM-P for 2 days±TO1317 (10 µM). Magnification: EM 2550x, Nile red (green) + nuclear Hoechst (red) staining: 60x. b) Effect of 250 µM-P±TO1317 on ^14^C-palmitate metabolism. Beta-cells were cultured for 24 h in the absence (white bars) or presence (gray bars) of 250 µM-P±TO1317, and subsequently incubated for 2 hrs with ^14^C-palmitic acid. Metabolism was measured as recovery of ^14^C-label in CO_2_, in lipids and lipid intermediates (pmol/2 h/10^3^ cells) and expressed as percentages of the control condition. Data shown as mean±se (n = 4), Student's t-test, ^$$^ P<0.01 and ^$$$^ P<0.001, versus control; * P<0.05 and ** P<0.01 versus palmitate.

In electron microscopy, beta cells exposed to 250 µM-P presented spindle-shaped empty-looking structures associated with the endoplasmatic reticulum (Figure 2a), and that have previously been associated with ER-stress and lipotoxicity [27], [28]; We noticed their presence in living cells, where their abundance seemed increased in the presence of TO1317. As demonstrated by Moffitt et al. [28], these structures correspond to lipid droplets in living cells when examined using light microscopy by vital nile-red staining; their number was markedly increased in the TO1317 condition (Figure 2a). These spindle structures and lipid droplets were not found in control cells cultured with or without TO1317 alone.

We assessed the effect of TO1317 on the cellular ability to transfer palmitate into cellular lipids, by measuring the incorporation of ^14^C-palmitate-derived carbon over a period of 2 hrs in cellular lipids, or as ^14^CO_2_-production, in control cells and in cells pre-exposed for 24hrs to 250 µM-P. Presence of 1 µM TO1317 stimulated the ^14^C-flux into lipids by 50±16% as compared to control (p<0.01) (Figure 2b). In contrast to the recent study by Green et al. [29], TO1317 was found to suppress ^14^C-palmitate oxidation in both the palmitate and the control condition. The apparent negative effect of 250 µM-P pre-exposure on ^14^CO_2_-formation is explained by a dilution effect on the ^14^C-label due to the increased intracellular palmitate levels, as shown before [7]. These observations support the view that channeling FA into stored neutral lipids correlates with protection against the cytotoxicity of cytoplasmic free acyl moieties and indicates the involvement of LXR in this process.

### Effect of LXR agonist on mRNA expression of desaturation enzymes in beta cells

**Figure 3:**
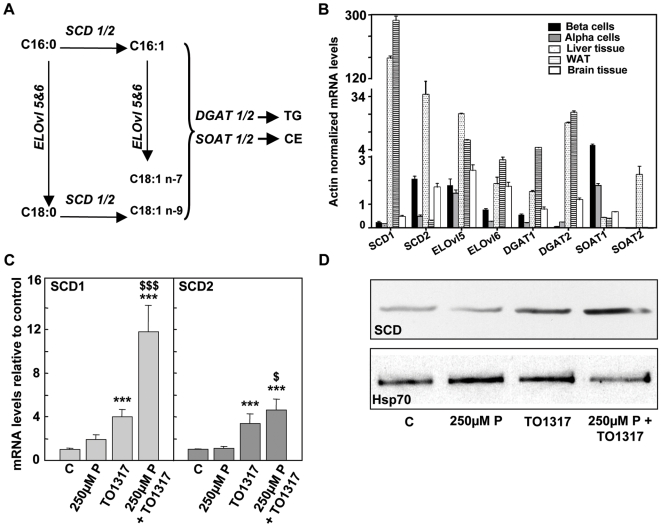
Enzymes involved in conversion of palmitate in response TO1317. a) Overview of enzymes involved in conversion of palmitate (C16:0) to palmitoylate (C16:1), vaccinate (C18:1 n-7), stearate (C18:0) and oleate (C18:1 n-9): stearoyl CoA desaturase (SCD1, SCD2), elongases (ELOvl5, ELOvl6), diacylglycerol transferase (DGAT1, DGAT2), sterol-O-acyl transferase (SOAT1, SOAT2). TG, triglycerides; CE, cholesteryl esters. b) qPCR result examining the mRNA levels of the above enzymes in freshly isolated rat beta cells, alpha-cells and liver, white adipose and brain tissue. Gene expression levels were normalized to actin, n = 3. c) qPCR analysis for SCD1 and 2, showing the effect of 8 days 250 µM-P±TO1317. Data shown as mean±sd, n = 4, *** p<0.001, compared to control; ^$^ p<0.05, ^$$$^ p<0.001, versus TO1317. d) Representative immunoblot showing SCD protein levels in beta cells cultured for 8 days with or without 250 µM-P±TO1317 (10 µM). Equal amounts of total protein were added on a 10% polyacrylamide gel. Representative for 4 independent experiments.

We examined the effect of TO1317 on mRNA expression of enzymes involved in fatty acid elongation (ELOvl 5 and 6), desaturation (SCD1 and 2) and esterification (DGAT1 and 2, SOAT 1 and 2) (Figure 3a). Since PPARα agonists have been previously found to induce beta cell protection through effects on enzymes that regulate palmitate metabolism [7], we compared the effects of the LXR agonist with those of the PPARα agonist combination clofibrate plus 9cis RA (Table 2).

**Table 2:** Effect of palmitate and LXR or PPARα agonists on gene mRNA expression levels calculated as ΔΔCt values compared to the indicated control conditions.

Genes	C16:0	TO1317	C16:0+TO1317	C16:0+clof/9-cis RA
	Compared to control	Compared to control	Compared to C16:0	Compared to C16:0
LXRα	1.4±0.3	2.5±1.3 *	1.3±0.7	1.7±0.6**
LXRβ	0.8±0.3	1.4±0.5	1.0±0.3	1.0±0.4
PPARα	0.7±0.2*	1.1±0.6	1.3±0.4	1.9±0.4 ***
FXR	0.7±0.1 *	1.0±0.1	1.1±0.4	1.6±0.5 **
SREBP1c	0.8±0.2	3.3±0.7 ***	3.5±1.2 ***	2.8±0.5 **
SCD1	0.5±0.2 ***	2.2±0.3 ***	2.5±0.6 ***	5.8±2.7 ***
SCD2	0.7±0.1 ***	1.8±0.3 **	2.4±0.3 ***	2.6±0.6 ***
ELOvl5	0.8±0.3	1.8±0.5	1.3±0.3	2.3±0.7 *
ELOvl6	0.8±0.1	1.9±0.6 **	1.0±0.3	2.4±0.8 *
GPAT	1.1±0.3	1.2±0.5	1.2±0.1	1.5±0.2 **
DGAT1	1.0±0.1	1.6±0.4	0.7±0.3	0.9±0.2
DGAT2	1.7±0.6	1.9±0.7 **	1.0±0.3	1.1±0.3
SOAT1	1.7±1.0	0.9±0.2	1.0±0.1	1.2±0.5
CPT1	1.9±0.6 **	1.2±0.1	0.6±0.2 *	1.9±0.2 ***
Acad l	1.4±0.7	0.8±0.1	0.7±0.2 *	1.5±0.2 **
Acad m	0.8±0.2	1.1±0.2	1.0±0.1	1.6±0.3 **
Acaa2	1.3±0.4	1.5±0.3	0.6±0.3 *	1.6±0.1 ***
Acaa1	0.8±0.2	1.9±0.5 *	0.8±0.4	1.6±0.1 ***
Acox1	1.0±0.3	1.2±0.2	0.7±0.3	1.6±0.4 **
Acox3	1.4±0.9	1.4±0.4	0.8±0.3	1.5±0.2 ***

Beta cells were exposed for 2 days to 250 µM-P±1 µM TO1317, or 250 µM clofibrate/2 µM 9-cis RA. qPCR values were normalized to actin and compared relative to the respective control conditions. Unpaired student t-test, two tailed, mean±SD, n = 4 – 6, * p<0.05, ** p<0.01, *** p<0.001.

When beta cells are compared with liver, brain, white adipose tissue (WAT) and endocrine alpha cells, they exhibit a high expression of SCD2, FA elongase-5 (ELOvl5) and sterol-O-acyl transferase-1(SOAT1), and a low expression of SCD1, ELOvl6, and the diacyl glycerol acyltransferase-1 and 2 (DGAT1 and DGAT2) (Figure 3b). Two days culture with 250 µM-P suppressed expression of SCD1 and 2 by, respectively 50 and 30% (Table 2); in presence of TO1317 these expressions were 5 and 3-fold higher than in its absence; a stimulation was also seen with the PPARα agonist combination (Table 2), but not observed in the presence of 9cis RA alone (results not shown). The effect of TO1317 on mRNA expression of SCD1 and SCD2 was accompanied by a similar effect on their protein expression (Figures 3c, 3d). The presence of 250 µM-P did not significantly alter the expression of the elongation and esterification enzymes (Table 2). It was however found to reduce the expression of the transcription factors PPARα and FXR, an effect that was counteracted by the presence of an LXR or PPARα agonist (Table 2). Both agonists also caused a marked increase in the expression of SREBP1c, which is compatible with the earlier reported role of SREBP1c in controlling SCD expression downstream of PPARα and LXR [22].

In contrast to PPARα agonists, the LXR agonist did not stimulate expression of enzymes involved in mitochondrial and peroxisomal oxidation. On the contrary, inhibitions up to 40% were measured (Table 2), which is compatible with the observed suppression of ^14^C-palmitate oxidation (Figure 2b).

### Inhibition of stearoyl-coenzyme A desaturase reverses the cytoprotective effects of LXR and PPARα agonists in beta cells

**Figure 4:**
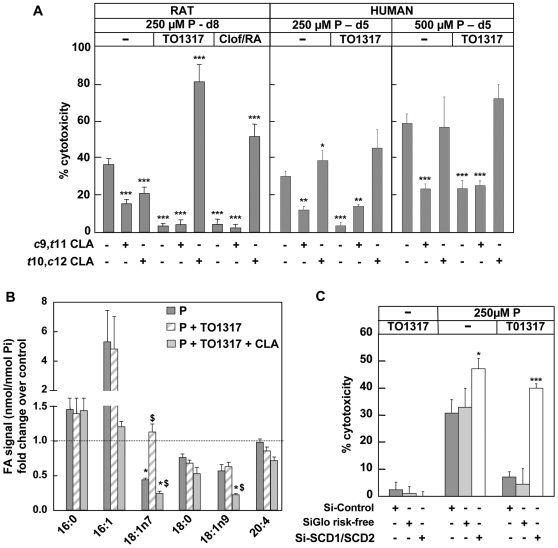
Δ9-desaturase activity plays a key role in protection against palmitate toxicity. a) Effect of inhibition of SCD. Rat and human beta cells were exposed to 250 µM-P or 500 µM-P±10 µM TO1317 or 250 µM clofibrate/2 µM 9RA, and/or *c*9,*t*11 CLA (40 µM), or *t*10,*c*12 CLA (40 µM). Percentage cytotoxicity shown as mean+sd, n = 3-4, *** p<0.001, versus palmitate. b) Effect of treatment on fatty acid composition as measured by GC-MS. Beta cells were exposed to 250 µM-P±10 µM TO1317 and±40 nM *t*10,*c*12 CLA for 8 days. The levels for C16:0, C18:0, C16:1, C18:1 and C20:4 measured as nmol/nmol Pi were expressed relative to their levels in control cells (ratios of saturated over monounsaturated FA are shown in Figure S1). Mean±sd, n = 4 - 6, * p<0.05 versus control, $ p<0.05 versus palmitate. c) RNA interference. Beta cells were transfected with a pool of siRNA's directed against SCD1 and 2 (Si-SCD1/SCD2), and exposed to 250 µM-P and/or TO1317 for 3 days. Si-control cells were treated with lipid transfection carrier (jetsi endo) only, or a pool of siGlo risk-free siRNA's (siGlo). Percentage cytotoxicity shown as mean+sd, n = 4, * p<0.05, *** p<0.001, versus Si-control cells.

In view of their effects on expression of SCD1 and SCD2 we examined whether the cytoprotective effects of the LXR and PPARα agonists were influenced by *t*10,*c*12-conjugated linoleic acid, a known inhibitor of SCD, or by its analog *c*9,*t*11-CLA, which has no effect on SCD activity [20]. Rat beta cells were cultured with 250 µM-P plus TO1317 for 8 days in the presence or absence of one of these compounds. The SCD-active CLA (*t*10,*c*12-CLA - 40 µM) not only prevented the protective effect of TO1317 but also augmented palmitate cytotoxicity, while no effect was seen with *c*9,*t*11-CLA (Figure 4a). This was also the case for the clofibrate-retinoic acid combination. None of the CLA-compounds were toxic by themselves, while they showed a mild protective effect on palmitate toxicity in the absence of TO1317 in rat beta cells. At this moment we can only speculate why both the active and the inactive CLA-isomers provided protection to palmitate in rat beta cells. *t*10,*c*12-CLA did not show this effect on human cells where it was - as expected - found to increase 250 µM-P toxicity in the presence, as well as in the absence of TO1317 (Figure 4a). Up to 5 different SCDs have been described in mammals with their own specific FA-preferences, whereas the effects of the CLAs have only been described for SCD1. It can thus not be excluded that other SCDs could be influenced by CLA treatment, and may affect palmitate toxicity. Our observations in rat beta cells indicate that the action of *t*10,*c*12-CLA on palmitate toxicity depends on the induction of SCD1 and SCD2 by TO1317, whereas cell survival in the absence of TO1317 does not depend on basal desaturase activity.

The SCD-active CLA (*t*10,*c*12 CLA) interfered with the effects of TO1317 on palmitate conversion as illustrated by GC-MS profiles of beta cells following culture. In the absence of palmitate and TO1317, beta cells exhibited comparable levels of C16:0, C18:0 (stearic acid) and arachidonic acid (C20:4), whereas their levels of C18:1 n-9 (oleic acid) and C18:1 n-7 (vaccenic acid) were lower, and those of C16:1 (palmitoleic acid) very low (Figure S1a). Culture with 250 µM-P led to a marked increase of the C16:1 fraction, and lowered C18:0 and C18:1 (n-7 and n-9) levels (Figure 4b). Consistent with a stimulatory effect on SCD, addition of TO1317 was found to normalize the C18:1 n-7 levels, but did not prevent the changes in C18:0, C18:1 n-9 and C16:1. The SCD-active CLA counteracted the effect of TO1317 on C18:1 n-7, and blocked formation of C16:1 and C18:1 n-9. No change was seen for the levels of C20:4 (arachidonic acid), a fatty acid derived from C18:2 (linoleic acid) through Δ5- and Δ6-desaturases, further indicating specific involvement of the Δ9 desaturase pathway. We noticed a higher C16:0 peak area after palmitate exposure, but it is impossible to discriminate whether this reflects uptake or adherence of the fatty acid. The effects of TO1317 and *t*10,*c*12 CLA on the ratios of saturated over monounsaturated fatty acids are shown in Figure S1b.

The role of SCD in beta cell protection against palmitate was further examined by RNA interference with this protein. Exposure of beta cells for 48 h to 50 nM siRNA-cationic lipid complexes targeted to both SCD1 and SCD2 resulted in a significant decrease in SCD-RNA levels (by 78±3% for SCD1, and 51±12% for SCD2 respectively), without affecting SREBP1c, LXR and PPARα mRNA levels (not shown). Jetsi-ENDO control and SiGLO risk free treated cells were used as control. Cell viability was not affected by the transfection protocol, and siRNA knock-down of SCD did not affect cell viability when compared to controls. Under these conditions, cells were taken 24 h after transfection and cultured for 72h in presence or absence of 250 µM-P with or without TO1317 (10 µM) (Figure 4c). SCD1/SCD2 siRNA-treated beta cells showed an increased palmitate toxicity (47±8%) as compared to control-transfected cells (Jetsi-ENDO control and SiGLO risk-free treated cells, p<0.05, n = 4) (Figure 4c). Moreover, protection by TO1317 was lost in SCD1/SCD2 siRNA-treated beta cells. Comparable results were obtained when cells were transfected with siRNAs directed to either SCD1 or SCD2 (data not shown). Cell viability was not affected by the transfection protocol, and siRNA knock-down of SCD did not affect cell viability when compared to controls.

### Interaction between desaturation pathway and palmitate induced ER stress

A causal relation between palmitate toxicity, ER stress and the accumulation of spindle-shaped structures in the ER has been suggested [27], [28], [30]. In our experiments these structures accumulated under protective conditions (See Figure 2). We therefore studied the expression of ER-stress markers in response to palmitate with, or without TO1317. Using this strategy we could, however, not establish a clear correlation between ER-stress, palmitate induced toxicity and protection against it (Table 3). When rat beta cells were exposed to 500 µM-P for 2 days – a condition with acute cell death- the expression levels of DNA-damage inducible transcript 3 (Ddit3/Chop) were found increased, in the absence, as well as in the presence of TO1317, while those of DnaJ homologue C3 (DnaJc3/P58) and heat shock 70 kDa protein 5 (HSPA5) remained unaffected (Table 3). No changes in expression for ER stress markers were found when cells were exposed to 250 µM-P with or without TO1317. We therefore further addressed this question by looking upstream from SCD. Δ9-desaturation requires electrons supplied via Ncb5or (NAD(P)H-cytochrome b5 oxidoreductase) [31]. Ncb5or was recently found to protect cells against palmitate induced ER-stress and lipotoxicity. Cells lacking Ncb5or^-/-^ show more signs of ER stress and a higher cytotoxicity in response to palmitate [32]. We found the expression levels of Ncb5or induced by TO1317 in presence of 250 µM and 500 µM-P, but not influenced by palmitate on its own (Table 3). These observations are consistent with a role of SCD to channel palmitate into less harmful pathways such as accumulation into neutral lipids, and -in our view- dissociate the accumulation of these lipids in the ER from apoptosis, since more of these spindle-shaped structures were formed under protective conditions.

**Table 3:** Effect of palmitate and LXR agonists on genes related to ER-stress.

Genes	TO1317	250 µM-P	250 µM-P+TO1317	500 µM-P	500 µM-P+TO1317
Ddit3	1.2±0.2	1.1±0.1	1.3±0.2	1.8±0.3 *	1.8±0.2 *
Dnajc3	0.7±0.1 *	1.0±0.2	0.7±0.02 *	0.8±0.2	0.8±0.1
Hsp5a	0.8±0.1	1.0±0.2	0.8±0.2	1.5±0.3	1.1±0.1
Ncb5OR	1.5±0.3	1.3±0.1	1.5±0.1 *	1.0±0.1	1.5±0.1 *

qPCR values normalized against actin and expressed relative to the control condition (vehicle treated). Cells were exposed for 2 days to 250 µM-P or 500 µM-P with or without 10 µM TO1317. Student t-test, two tailed, mean±sd, N = 3 – 6, * p<0.05, ** p<0.01.

### LXR isotype specificity in modulation of palmitate toxicity through SCD and SREBP1

**Figure 5:**
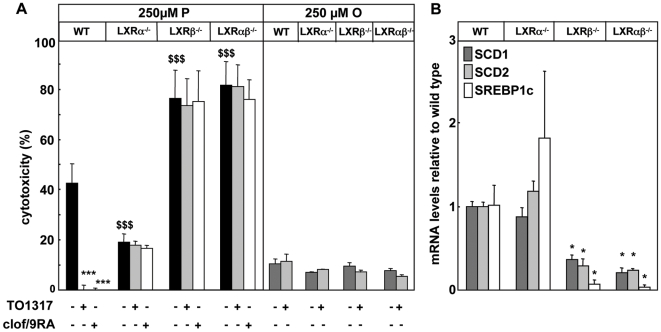
Effect of LXR and PPARα agonists on palmitate toxicity in LXR KO beta cells. Primary mouse beta cells were exposed to a) 250 µM-P or 250 µM oleate for 8 days in presence or absence of 1 µM TO1317 or 250 µM clofibrate/2 µM 9-cis RA. Percentages of cytotoxicity are shown as mean+sd, n = 3, *** p<0.001, TO1317 and clofibrate/9RA versus palmitate; ^$$$^ p<0.001, palmitate toxicity in LXR^-/-^ as versus wild type. b) SCD1, SCD2 and SREBP1c mRNA levels in LXR^-/-^ islets as compared to wild type. Q-PCR signals shown as mean+sd, n = 3, * p<0.001.

The cytoprotective effects of LXR and PPARα agonists were measured in beta cell preparations isolated from LXRα^-/-^, LXRβ^-/-^, LXRαβ^-/-^ and wild type mice. Beta cell survival in control conditions was similar for the 4 genotypes and was not influenced by the presence of TO1317 or clofibrate/9-cis RA. Culture with 250 µM-P was cytotoxic for the four preparations (Figure 5a). Beta cells from LXRβ^-/-^ and LXRαβ^-/-^ mice showed a much higher susceptibility to palmitate-toxicity (almost 100% cell death) than wild type cells, whereas those from LXRα^-/-^ mice were less susceptible. No differences in oleate toxicity were seen (Figure 5a). The same observations were made when cells were exposed to 100 µM-P, leading to almost 70% cell death in absence of LXRβ (results not shown). Beta cells from wild-type mice were strongly protected by the LXR-agonist TO1317, as well as by clofibrate/9-cis RA. This was not the case for LXRα^-/-^, LXRβ^-/-^ and LXRαβ^-/-^ (Figure 5a). These observations indicate that the protective action of PPARα agonists on palmitate toxicity depend on the presence of both LXRα and β and downstream signaling pathways.

We also conducted the above-described mRNA analysis on islets isolated from LXRα^-/-,^ LXRβ^-/-^, LXRαβ^-/-^ and wild type mice (Table S2). The islets did not present obvious differences in cellular composition with more than 65% percent beta cells in each preparation (results not shown). No significant differences were noticed between islets from wild-type mice and from LXRα^-/-^mice, while absence of LXRβ was associated with a markedly reduced expression of SCD1 and 2, and of SREBP1c (Figure 5b), and higher expression of DGAT1, SOAT1, and ELOvl5 (Table S2). This observation is compatible with a key role of LXRβ in the expression of SCD and SREBP1c, and their influence on the cellular susceptibility to palmitate toxicity and further supports a role of SREBP1c downstream of LXR < PPARα [33].

### Correlation between SCD expression in isolated beta cells and their susceptibility to palmitate

The view that SCD-expression in beta cells may vary with their environmental conditions in vivo, led us to explore three conditions in which rat beta cells could be expected to express higher SCD-levels after isolation. These preparations were then analyzed for their susceptibility to palmitate toxicity.

**Figure 6:**
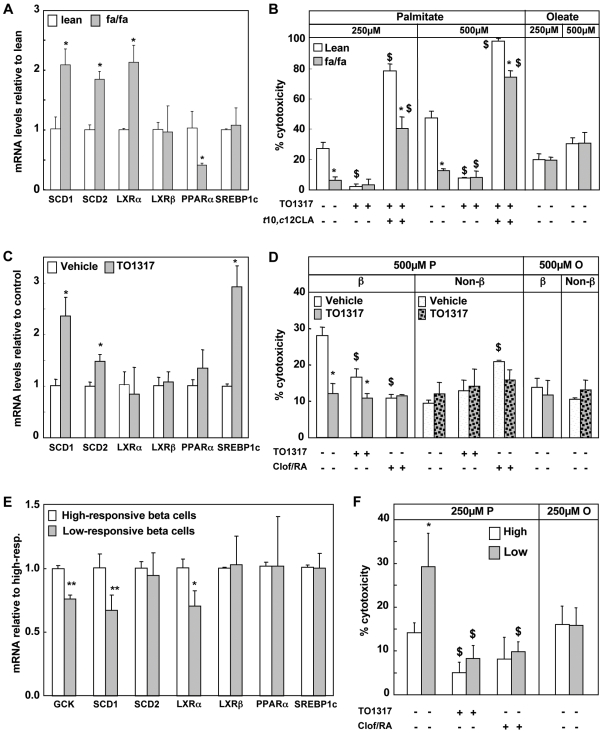
SCD levels regulate beta cell susceptibility for palmitate in vivo. Beta cells and alpha-enriched cell preparations were obtained from: a-b) Zucker fatty (fa/fa) and lean control rats (fa/-) and exposed for 8 days to 250 µM and 500 µM palmitate or oleate in the presence or absence of 10 µM TO1317±40 nM *t*10,*c*12 CLA. Percentages of cytotoxicity are shown as mean±sd, n = 3, * p<0.001 fa/fa versus lean control, $ p<0.001 versus palmitate; or isolated from c-d) Wistar rats treated by oral gavages for 5 days with 40mg/kg BW TO1317 or vehicle (DMSO/PBS, 1/3), and exposed for 2 days to 500 µM palmitate or oleate in the presence or absence of 1 µM TO1317 or 250 µM clofibrate +2 µM 9-cis RA. Results are shown as mean±sd, n = 3, * p<0.001 TO1317-cells versus vehicle, $ p<0.001 versus control; or e-f) Beta cells were FACS sorted based on their glucose-induced increase in [NAD(P)H]-autofluorescence into two subpopulations characterized by a high or low glucose-responsive phenotype [8], and exposed for 8 days to 250 µM palmitate or oleate in the presence or absence of 1 µM TO1317 or 250 µM clofibrate plus 2 µM 9-cis RA. Percentages of cytotoxicity are shown as mean±sd, n = 3, * p<0.01 low versus high NAD(P)H-cells; $ p<0.01 agonist treatment versus control. a,c,e) Q-PCR analysis showing the differences in expression levels of glucokinase, SCD1, SCD2, LXRα, LXRβ, PPARα and SREBP1c. Results are shown as mean±sd, n = 3-7, * p<0.05, ** p<0.01 versus the respective control preparations.

*The first condition* is represented by beta cells isolated from Zucker fatty rats lacking leptin receptor (fa/fa). Since leptin suppresses SCD [34] these animals show increased Δ9-desaturation activities and decreased saturated/mono-unsaturated fatty acid ratios in serum and tissues, including pancreas [35]. Compare to their lean controls (fa/-), beta cells from fa/fa animals showed 2-fold higher mRNA levels for SCD1, SCD2; their LXRα expression was also elevated, whereas PPARα expression was suppressed (Figure 6a), Table S3. Beta cells isolated from fa/fa animals showed a significantly lower susceptibility to palmitate-induced cell death than those isolated from lean controls (fa/-): after 8 days the cytotoxicity of 250 µM-P and 500 µM-P was, respectively, 6±2% and 13±1% for in fa/fa versus 28±4% and 48±5% in the fa/- controls (Figure 6b). In contrast, the cytotoxicity of oleate was low and similar in both preparations. When the LXR-agonist TO1317 was added to the palmitate conditions, it gave a mild reduction of palmitate toxicity in fa/fa beta cells, whereas fa/- cells were efficiently protected. Protection was counteracted by the SCD inhibitor *t*10,*c*12 CLA which even amplified the palmitate toxicity in both cell preparations. Beta cell vitalities of cells isolated from lean and Fatty Zucker rats were similar in control conditions. These data indicate that differences in SCD expression and activity in beta cells have consequences for their susceptibility to saturated fatty acids.

*The second condition* was experimentally induced by treatment of rats with the LXR agonist TO1317 (40 mg/kg body weight by oral gavage for 5 days) or with a control vehicle. The pancreases were then removed for purification of beta cells and alpha cell enriched fractions by FACS-sorting. Beta cells from TO1317-treated rats showed elevated SCD1, SCD2 and SREBP1c mRNA levels, and similar levels of LXRα, LXRβ and PPARα (Figure 6c). Their palmitate susceptibility was lower after 500 µM-P -2 days but could not be further reduced by addition of LXR or PPARa agonists, while these compounds induced protection in the vehicle-derived beta cells. Cells from TO1317-treated and vehicle-treated animals showed the same low oleate cytotoxicity (Figure 6d). Comparable data were obtained after culture at 250 µM-P for 8 days (cytotoxicity of 10±3% in TO1317-cells versus 30±1% in vehicle-cells). These observations indicate that in vivo treatment with the LXR-agonist has induced a beta cell phenotype with a sustained protection against palmitate. In prior work we showed that clof/9-cis RA did not influence palmitate toxicity in alpha-cell enriched preparations [7]. In the present study alpha-cell enriched preparations from TO1317- or vehicle-treated rats exhibited the same palmitate toxicity; this was also the case when TO1317 was added to the culture medium. No differences were seen in the viability of the beta cell or alpha cell-enriched preparations isolated from TO1317- or vehicle-treated rats and cultured in the control condition

In prior work we have shown that primary beta cells can be less susceptible or even resistant to palmitate toxicity, and that this property is heterogeneously expressed [7]. To explore this observation, *and as a third condition*, we have separated freshly isolated rat beta cells into two subpopulations according to their cellular metabolic responsiveness to an acute 7.5 mM glucose stimulation, as described in prior studies by our group [8], [36]. The high-responsive beta cells were found to express higher levels of SCD1 and LXRα than the low-responsive beta cells; their higher expression of glucokinase was confirmed and served as an index for their higher metabolic responsiveness (Figure 6e) [37]. They also presented a lower cytotoxicity after 8 days culture with 250 µM-P (Figure 6f), or 2 days with 500 µM-P (data not shown). Addition of the LXR- or PPARα-agonists exerted a much more pronounced protective effect in the low-responsive subpopulation, which is in part related to their higher palmitate toxicity but which also indicates that they can generate cytoprotective mechanisms when receiving extracellular signals. As with the previously described conditions, the two tested cell populations underwent a similar low oleate cytotoxicity.

## Discussion

The mechanisms regulating the balance between functional adaptation, lipid accumulation, dysfunction, or beta cell death in response to chronic elevated FA levels are poorly understood. Clarifying these mechanisms could help to develop new strategies to prevent beta cell deterioration in type 2 diabetes. Several studies have reported the potential of PPAR ligands to preserve functional beta cell mass in obesity models and in clinical type 2 diabetes [38], [39], but only a limited number of reports has characterized the role of LXRs in this context [25], [26]. LXR activation has been shown to stimulate insulin secretion in vitro via de novo lipid synthesis [26], [29], [40], while in vivo LXR agonists are shown to reduce serum glucose levels and to improve glucose tolerance and insulin resistance in obesity models [41], [42].

**Figure 7:**
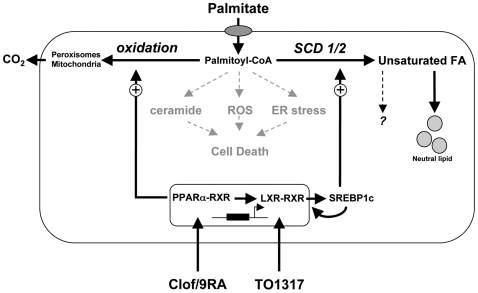
Hypothetical lipoprotection model using LXR or PPAR ligands. Long chain saturated FA, such as palmitate, induce cell death through reactive intermediates, ceramide generation or via ER stress. Activation of LXR downstream of PPARα activates SREBP1c and induces transcription of genes encoding SCD1 and SCD2. This results in an increased formation of monounsaturated FA and directs the flow of palmitate towards intracellular storage as neutral lipids. Next to inducing SCD levels, activation of PPARα results in an increased turn-over of FA by stimulation of β-oxidation, with detoxifying effects on both saturated and monounsaturated FA.

We have used primary beta cell preparations to examine ways to induce protection against beta cell death during prolonged exposure (up to 16 days) to elevated concentrations of the saturated fatty acid palmitate. Protection was seen with PPARα agonists and correlated with an increased oxidation rate and an increased expression of genes involved in β-oxidation [7], as well as in Δ9-desaturation and lipogenesis. In view of the prior reported protective action of SCD [19]-[21], which is mainly a LXR target gene, we now demonstrate that both PPARα and LXR-agonists provide long-term protection in rodent and human beta cells through a pathway involving Δ9-desaturation. We propose the following model (schematic overview in Figure 7): Δ9-desaturase expression in beta cells is regulated by LXR. Direct activation of LXR, or induction of LXR downstream of PPARα regulates SCD-transcription, possibly in a SREBP1c-dependent way. As a result exogenous palmitate is desaturated, elongated and more efficiently channeled to neutral storage pools associated with detoxification. This view is supported by the observation that TO1317 confers protection to saturated, but not to monounsaturated FA; and by the increased toxicity of palmitate upon SCD inhibition, or by its silencing. SREBP1c, known to regulate ELOvl6 (which preferentially elongates C16>C18), as well as SCD-expression [29], [43], [44], GPAT and DGAT2, both involved in triglyceride formation, were all induced in response to both LXR and PPARα ligands, consistent with an accumulation of palmitate into intracellular lipid pools [29], [43], [45]. Our observations unambiguously indicate the involvement of SCD in this process but do not exclude that protection against palmitate could lay at a step between fatty acid desaturation and incorporation of unsaturated fatty acids into neutral lipids. Deposition into neutral lipids was not essential to induce SCD-protection in Min6 cells [20]. Newly formed monounsaturated FA could exert specific protective effects [46].

Absence of LXR abolished protection to palmitate by TO1317, as well as by clofibrate/9-cis RA. This observation indicates that PPARα modulates LXR-expression in beta cells, as already shown for other cell types [47]. PPARα agonists were indeed found to induce LXRα mRNA levels. These findings further imply that PPARα-induced protection to saturated FFA largely depends on LXR-regulated SCD and not on its stimulatory action on fatty acid oxidation. In contrast to LXR, ligands for PPARα however not only detoxify saturated [48], but also monounsaturated FFA [7], indicating that an increased oxidation rate contributes to gain protection. Islets from mice lacking LXRβ showed a strong suppression of SCD and SREBP1c correlating with an increased susceptibility to palmitate, but not oleate, which further emphasizes the importance of Δ9-desaturation.

Although the expression levels of LXRα seem to correlate with resistance to cytotoxicity, our results using LXR knock-out mice imply that both LXRα and LXRβ are required to gain protection by LXR or PPAR ligands, whereas LXRβ seems to control basal transcription from the SCD-promoter via SREBP1c. These results point to a complex regulation of the SCD promoter in response to LXRs, PPARs and SREBP1c. Comparable observations, where both LXR subtypes are required to control transcription in concert with PPAR and/or SREBP1c, have been reported for Elovl5, Δ6-desaturase and SREBP-1c [33], [49].

In the proposed model SREBP1c seems to play a central role downstream from PPARα and LXR. SREBP1c activation has already been implicated in the adaptive changes that underlie beta cell hypersecretion in response to elevated glucose [26], [29], [50]. In contrast however, hyper-activation of SREBP1c by its over expression or its activation by glucose and/or palmitate, has been linked to glucolipotoxicity, suppression of glucose-stimulated insulin secretion (GSIS), and was proposed as a mechanism to explain islet failure in Zucker Diabetic fatty rats (ZDF) [51]-[56]. Inactivation of SREBP1c in these islets, however, failed to normalize GSIS, thereby excluding SREBP1 and triglyceride-accumulation as the main cause behind defective secretion in this model [57]. Consistent with a role for SREBP1c in controlling insulin secretion, TO1317 has been shown to stimulate GSIS and insulin biosynthesis in beta cell lines and mouse islets via a stimulation of glucose and lipid metabolism [26], [29]. Negative effects of TO1317 on cellular vitality and/or beta cell proliferation were however also reported and need attention when LXR agonists would be considered to treat type 2 diabetes [45], [58], [59]. We therefore examined TO1317±palmitate for a period up to 16 days; no adverse effects on cell viability and cell numbers were observed, and palmitate toxicity was suppressed by an average of 88% during the whole period. In this context it also needs to be mentioned that TO1317 was recently found, in parallel to SCD1, to induce CPT1 mRNA levels and to stimulate β-oxidation in INS1-cells [29], and in hepatocytes [60], whereas TO1317 showed the opposite effects in our experiments using primary rat beta cells. These divergent results could relate to differences in experimental protocols such as FFA concentrations, FFA/BSA ratio's, use of serum, or relate to differences in levels and duration of LXR-activation, but also point to differences between beta cell lines and primary cells. Lai et al. [61] recently postulated that the lower sensitivity of human beta cells and Min6 cells to palmitate toxicity as compared to INS-I cells, could be related to higher SCD1 levels. In our experiments however, comparable cytotoxic responses to palmitate (250 and 500 µM) were found for FACS-purified rat, mouse and human beta cells. Comparison of the expression profiles of primary cells and cell lines in response to FA and agonists might reveal differences explaining these divergent observations. Cross talk between PPAR- and LXR-levels and competition to bind RXRs or with other nuclear receptors could play a role [62].

Busch et al. reported that palmitate-resistant Min6 cells were mainly characterized by an increase of SCD1 [20]. Our study cannot discriminate between the respective contribution of SCD1 or SCD2. Whereas SCD2 was more prevalent in freshly isolated beta cells, SCD1 was induced more potently by LXR and PPARα. SCD1 contains SREBP1c and LXR-responsive elements in its promoter region, whereas its expression is actively repressed by leptin [63], [64]. Activation of SCD in animals lacking normal leptin signaling can thus be predicted to lower the cellular susceptibility to toxic palmitate concentrations. This hypothesis is supported by our observations on beta cells isolated from TO1317-treated animals and from Zucker fatty rats (lacking a functional leptin receptor), which were protected from palmitate but not oleate toxicity, and by a rise of palmitate toxicity in the presence of an SCD-inhibitor. The importance of SCD in beta cells is also documented by the loss of SCD1 in leptin^ob/ob^ mice, leading to an accelerated progression to overt diabetes [65]. Absence of SCD in this model was accompanied by appearance of a morphologically distinct class of dysfunctional islets showing a suppression of PPARα and its target genes, increased triglycerides, FFA, and higher levels of saturated FA than a second class of islets displaying normal features [65]. These observations are also interesting in view of the differences in palmitate susceptibility and SCD1 expression levels observed in the high and low glucose-responsive beta cells, and in view of our prior work where it was shown that primary beta cells show differences in susceptibility or can even be resistant to palmitate toxicity [7]. Recent observations in leptin receptor-overexpressing obese db/db mice showed suppressed SREBP1c and adipogenic gene-expression levels, severe diabetes and beta cell loss 6 weeks before the db/db controls [66]. These findings support the idea that the inherent adipogenic phenotype of individual beta cells via chain elongation and desaturation provides the cells with a protective mechanism to cope with elevated FFA. Our observations contain an important message since SCD1-inhibitors are evaluated to treat obesity and the related metabolic syndrome [67], [68]. Although suppression of SCD1 might show beneficial effects on adiposity, these benefits may come at the expense of pancreatic beta cells. Clinical studies already indicated an increased risk to develop diabetes in predisposed obese patients using a mixture of c9,t11 and t10,c12 CLA for weight management [69], [70].

In conclusion, activation by PPAR and LXR agonists of a pathway controlled by LXR involving Δ9-desaturation, and chain elongation followed by esterification leads to detoxification of saturated FA and prevents beta cell death.

## Methods

### Materials

Chemicals and FA were purchased from Sigma-Aldrich (Bornem, Belgium). Stock solutions of FA were prepared in 90% ethanol. Stock solutions of clofibrate, TO1317, PCN and 9-cis RA were dissolved in absolute ethanol or DMSO. [U-^14^C]-palmitic acid was obtained from Perkin Elmer Life Sciences (Zaventem, Belgium).

### Ethics statement

Animal experiments were approved by the local Ethical Committee for Animal Experimentation of the Vrije Universiteit Brussel. All manipulations were carried out in accordance with the European Community Council Directive (86/609/EEC). Human endocrine cells were obtained from the Brussels-Beta Cell Bank. Human pancreata from donors at European hospitals affiliated with Eurotransplant Foundation (Leiden, The Netherlands) are processed by the Beta-Cell Bank of the Juvenile Diabetes Research Foundation Center for Beta Cell Therapy in Brussels with the purpose of preparing islet cell grafts for clinical trials. Isolated fractions that do not fulfill the quality criteria for transplantation, can be made available to research projects approved by the Medical Ethics Committee of the University Hospital (UZ Brussel-Vrije Universiteit Brussel). Approval to use beta cells for this project was obtained from the Medical Ethics Committee of the UZ Brussels – Vrije Universiteit Brussel.

### Isolation and culture of beta cell preparations

Pancreatic islets were isolated from 10 wks old male Wistar and Zucker rats (lean and fa/fa) and male LXR knock-out mice (8 to 12 wks),. LXRαα^–/–^, LXRβ^–/–^, and LXRααβ^–/–^ mice were backcrossed from a 129/Sv to a C57BL/6 background for at least ten generations [71]. Rat and mouse islets were dissociated into single cells and purified by FACS into beta cells (mean purity 90% insulin-positive cells) and alpha enriched-cells (75% glucagon-positive cells, 25% insulin-positive cells) using cellular light-scatter and FAD-autofluorescence as discriminating parameters. The methods for rodent islet isolation, dissociation and FACS purification of islet beta and alpha cells have been described previously [72]. In one set of experiments, rat beta cell subpopulations with, respectively, high and low glucose-responsiveness were FACS sorted using glucose-induced increases in cellular level of NAD(P)H-autofluorescence as discriminator, as described in our previous studies reporting on the identity and function of these cells [8], [10], [37]. Human endocrine cells were obtained from the Brussels-Beta Cell Bank and prepared as described by Ling and Pipeleers [73]. These endocrine fractions were enriched by FACS to a mean purity of 60% beta cells and further enriched under culture conditions favoring beta cell survival.

For cell viability experiments, beta cells were cultured in polylysin-coated microtiter plates with Ham's F10 containing 10 mM glucose, 1% charcoal-extracted BSA (fraction V, radioimmunoassay grade, Boerhinger-Mannheim), 2 mM L-glutamine, 50 mM 3-isobutyl-1-methyl-xanthine, 0.075 mg/ml penicillin and 0.1 mg/ml streptomycin. Test reagents were added, with controls receiving similar dilutions of solvent. The unbound FFA concentrations in the presence of 1% albumin were calculated to correspond to 9 and 27 nM after addition of 250 and 500 µM palmitate using a multiple stepwise equilibrium model using the association constants for binding of palmitate to the first six binding sites of albumin, as described by Richieri et al. [74].

Media were refreshed every 2 to 3 days. The percent living and dead cells was determined as previously described and toxicity indices calculated [6]. For metabolic and gene expression studies, freshly isolated cells were re-aggregated and cultured in suspension [72].

Samples for electron microscopy were fixed in cacodylate-buffered glutaraldehyde (4.5%, pH 7.3), postfixed in osmium tetroxide (1%) and embedded in Spurr's resin. Ultrathin sections were stained with uranylacetate and lead citrate and examined on a Zeiss EM 109 electron microscope. Nile red (Molecular probes, Invitrogen) was used to detect lipid accumulation in living cells (axioplan, Zeiss).

Palmitate oxidation was measured in KRBH medium containing 0.2% BSA, 2 mM Ca^2+^, 10 mM hepes and 0.5 µCi [U-^14^C]-palmitic acid, and 250 µM unlabeled palmitic acid, as described prior [7]. The rate of [U-^14^C]-palmitic acid oxidation was assessed through formation of ^14^CO_2_ [75].

### LXR Agonist Treatment

Male Wistar rats (approx. 250 g), were treated with 40 mg/kg body weight/day of LXR agonist TO1317 (Sigma–Aldrich) dissolved in DMSO/PBS (1∶3), given daily at the onset of the dark cycle by oral gavages for 5 days. Control rats were given only vehicle. After 5 days beta cells and alpha-enriched cells were FACS-purified and used for viability assays as described.

### siRNA Transfection

Transfection was conducted in freshly isolated rat beta cells. Cells were aggregated in presence of 50 nM siRNA-lipid complexes using Jetsi-ENDO (Polyplus, France) as transfection agent [76]. siRNA's for rat SCD1 and SCD2 were siGENOME SMART pool reagents (J-096598-00 and L-099222-01; Dharmacon), pools of four different siRNAs. Control cells were treated with Jetsi-ENDO only or transfected with non-targeting siGLO RISC-free (D-001600-01). Transfection did not result in cytotoxicity. After 24 h, aggregates were dissociated to single cells. Half of the cells were used in the toxicity assay with an additional 72 h culture; the other half was reaggregated and RNA extracted 24 h later. Over 95% of reaggregated siGLO-transfected beta cells showed cytoplasmic siRNA-associated fluorescence.

### Gene, protein and lipid analysis of beta cell aggregates

RNA was extracted using RNeasy columns (Qiagen). RNA quality was verified by Agilent Bioanalyzer. Following removal of genomic DNA (TURBO DNA-free, Ambion, Austin, Texas, U.S.A.) and reverse-transcription (High-Capacity cDNA Archive Kit, Applied Biosystems, Foster City, U.S.A.), targets were amplified on ABI Prism 7700 using TaqMan Universal PCR Master Mix and specific TaqMan MGB probes (Applied Biosystems, assays' IDs available on request). Expression levels of target genes were normalized to β-actin or 18S (ΔCt) and expressed versus a chosen calibrator (comparative ΔΔCt method).

Beta cell aggregates were lysed in RIPA buffer containing protease inhibitors (Sigma). Proteins were resolved by SDS-PAGE on 10% (w/v) acrylamide:bisacrylamide gels and transferred to nitrocellulose membranes using iblot (Invitrogen, Belgium). Immunodetection of SCD was performed using mouse anti-SCD1/2 antibodies (Abcam). Hsp70 served as housekeeping control (Santa Cruz Biotechnology, CA, USA). Horseradish peroxidase-linked secondary antibodies (Santa Cruz Biotechnology) were used. Immunoreactive proteins were visualized by enhanced chemiluminescence (ECL, Pierce).

Lipid extracts were prepared from beta cell aggregates, fortified with 10 nmol tricosanoic acid (IS), dried and treated with 0.5 ml 0.5 N HCl in 90% acetonitrile at 100°C for 1 hr [77]. After adding water, released FA were extracted in 2x1 ml hexane, and an aliquot of the aqueous phase was analyzed for phosphate to estimate phospholipid content. The hexane extract was dried, silylated with N-tert butyldimethylsilyl-N-methyltrifluoroacetamide with 1% tert butyldimethylchlorosilane (Pierce)/pyridine (1/1, v/v) and analyzed by GC-MS (Trace GC-MS, Thermo Finnigan), equipped with an automated cold-on-column injector and high oven temperature device connected to a BPX70 column (30m×0.25 mm; 0.25 µm). Fatty acids were identified by comparing elution times to standards and by their mass spectrum. Total ion current signals, obtained in EI^+^ mode at 35 eV, were related to the IS signal and converted to nmol fatty acid using relative response factors by analyzing a reference fatty acid mixture containing tricosanoic acid (GLC-538, free acids; Nu-Chek Prep), derivatized like the extracts.

### Data analysis

Data are presented as means±SD of n independent experiments. Single comparisons were performed by the Student's paired *t*-test. For multiple comparisons, data were analyzed by ANOVA, and group comparisons using Student's paired or unpaired *t* test, with correction of the *P* values for multiple comparisons by the Bonferroni method.

## Supporting Information

Figure S1 Effect of inhibition of SCD on GC-MS fatty acid profiles. Beta cells were exposed for 8 days to 250 µM-P±1 µM TO1317±t10,c12 CLA (40 µM). Lipid extracts were prepared and analyzed by GC-MS as described. a) Representative chromatograms, covering the elution of C14:0 till C22:6 Figure representative for 6 independent analyses. b) The ratios between the saturated and monounsaturated FA were calculated. Mean±SE, n = 5 - 6, * p<0.05, ** p<0.01 versus control, $ p<0.05, $$<P 0.01 versus palmitate
(1.47 MB TIF)

Table S1 Effect of TO1317 on cytotoxicity of FA with different chain length and saturation. Beta cells were exposed for 2 or 8 days to the indicated FA±1 µM TO1317. Mean±SD (n>4), # P<0.001 toxicity of different FA compared to C16:0; * P<0.05, *** P<0.001, the TO1317 condition compared to the respective control FA condition.
(0.04 MB DOC)

Table S2 mRNA expression levels in islets from LXR KO mice relative to wild type. qPCR values normalized against 18S and compared relative to the expression levels in wild type islets. Unpaired student t-test, two tailed, mean±SD, n = 3, Differences were considered significant with p<0.01. ** p<0.01 and *** p<0.001, compared to wild type control. ND = not detected
(0.05 MB DOC)

Table S3 mRNA expression levels in beta cells from Zucker obese rats relative to lean controls. qPCR values normalized against β-actin and expressed relative to the expression levels in beta cells from Zucker lean controls. Unpaired student t-test, two tailed, mean±SD, n = 3, Differences were considered significant with ** p<0.01 and *** p<0.001.
(0.05 MB DOC)
