# Supplementary figures and images for: Susceptibility of Pancreatic Beta Cells to Fatty Acids Is Regulated by LXR/PPARα-Dependent Stearoyl-Coenzyme A Desaturase
Source: PLoS One. 2009 Sep 29;4(9):e7266. doi: 10.1371/journal.pone.0007266 (PMC2746288; doi:10.1371/journal.pone.0007266)

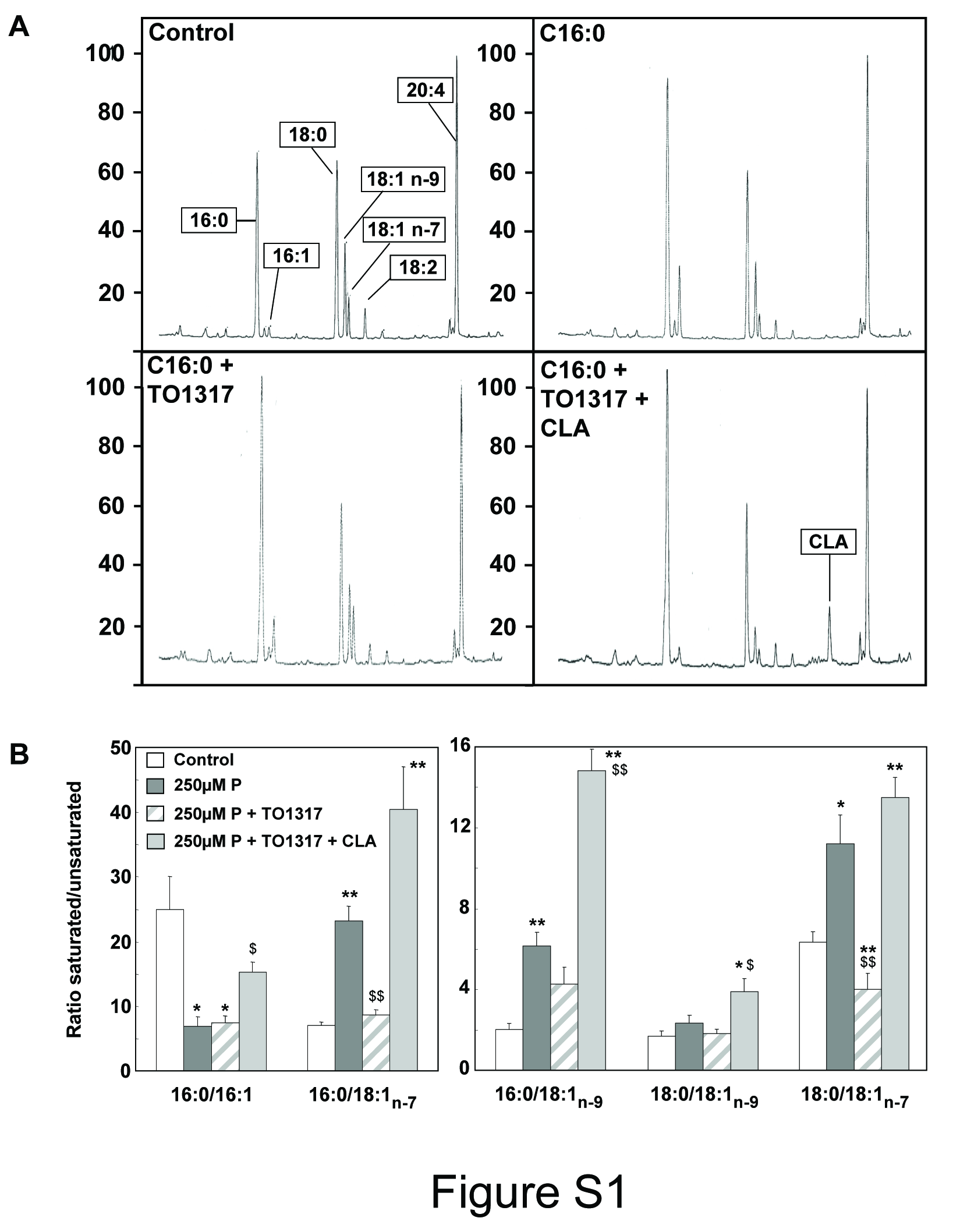

Supplement: Figure S1 — Effect of inhibition of SCD on GC-MS fatty acid profiles. Beta cells were exposed for 8 days to 250 µM-P±1 µM TO1317±t10,c12 CLA (40 µM). Lipid extracts were prepared and analyzed by GC-MS as described. a) Representative chromatograms, covering the elution of C14:0 till C22:6 Figure representative for 6 independent analyses. b) The ratios between the saturated and monounsaturated FA were calculated. Mean±SE, n = 5 - 6, * p<0.05, ** p<0.01 versus control, $ p<0.05, $$<P 0.01 versus palmitate (1.47 MB TIF) [file pone.0007266.s001.tif]
