# Supplementary material for: Susceptibility of Pancreatic Beta Cells to Fatty Acids Is Regulated by LXR/PPARα-Dependent Stearoyl-Coenzyme A Desaturase
Source: PLoS One. 2009 Sep 29;4(9):e7266. doi: 10.1371/journal.pone.0007266 (PMC2746288; doi:10.1371/journal.pone.0007266)
Supplement: Table S1 — Effect of TO1317 on cytotoxicity of FA with different chain length and saturation. Beta cells were exposed for 2 or 8 days to the indicated FA±1 µM TO1317. Mean±SD (n>4), # P<0.001 toxicity of different FA compared to C16:0; * P<0.05, *** P<0.001, the TO1317 condition compared to the respective control FA condition. (0.04 MB DOC) [file pone.0007266.s002.doc]

Table S1: Effect of TO1317 on cytotoxicity of FA with different chain length and saturation.

|  | ***Percent cytotoxicity*** | | |
| --- | --- | --- | --- |
| **FA** | **500 µM – 2 days** | **250 µM – 8 days** | **500 µM – 8 days** |
| C16:0 | 40 ± 12 | 41 ± 1 | 77 ± 11 |
| + 1µM TO1317 | **13 ± 3 ***** | **7 ± 3 ***** | **32 ± 1 ***** |
| C16:1 | 16 ± 3 ^#^ | 22 ± 3 ^#^ | 30 ± 2 ^#^ |
| + 1µM TO1317 | 14 ± 4 | 23 ± 4 | 32 ± 0.5 |
| C18:1, n-7 | 16 ± 5 ^#^ | 29 ± 1 ^#^ | 47 ± 3 ^#^ |
| + 1µM TO1317 | 16 ± 7 | 26 ± 4 | **37 ± 4 **** |
| C18:0 | 54 ± 7 | 38 ± 8 | 89 ± 6 |
| + 1µM TO1317 | 49 ± 8 | **17 ± 3 ***** | **75 ± 1 **** |
| C18:1, n-9 | 19 ± 7 ^#^ | 22 ± 5 ^#^ | 37 ± 2 ^#^ |
| + 1µM TO1317 | 17 ± 4 | 24 ± 6 | 33 ± 2 |

Beta cells were exposed for 2 or 8 days to the indicated FA ± 1 µM TO1317. Mean ± SD (n > 4), ^#^ P < 0.001 toxicity of different FA compared to C16:0; * P < 0.05, *** P < 0.001, the TO1317 condition compared to the respective control FA condition.
