# Supplementary material for: Susceptibility of Pancreatic Beta Cells to Fatty Acids Is Regulated by LXR/PPARα-Dependent Stearoyl-Coenzyme A Desaturase
Source: PLoS One. 2009 Sep 29;4(9):e7266. doi: 10.1371/journal.pone.0007266 (PMC2746288; doi:10.1371/journal.pone.0007266)
Supplement: Table S2 — mRNA expression levels in islets from LXR KO mice relative to wild type. qPCR values normalized against 18S and compared relative to the expression levels in wild type islets. Unpaired student t-test, two tailed, mean±SD, n = 3, Differences were considered significant with p<0.01. ** p<0.01 and *** p<0.001, compared to wild type control. ND = not detected (0.05 MB DOC) [file pone.0007266.s003.doc]

Table S2: mRNA expression levels in islets from LXR KO mice relative to wild type.

| **Gene** | **LXRα^-/-^** | **LXRβ^-/-^** | **LXRαβ ^-/-^** |
| --- | --- | --- | --- |
| LXRα | ND | 0.8 ± 0.4 | ND |
| LXRβ | 1.2 ± 0.4 | ND | ND |
| PPARα | 1.1 ± 0.5 | 1.0 ± 0.3 | 1.2 ± 0.3 |
| FXRα | 1.8 ± 0.5 | 2.0 ± 0.5 | 2.1 ± 0.4 |
| SREBP1c | 1.8 ± 0.8 | **0.1 ± 0.05 ***** | **0.03 ± 0.01 ***** |
| SCD1 | 0.9 ± 0.1 | **0.4 ± 0.06 ***** | **0.2 ± 0.06 ***** |
| SCD2 | 1.2 ± 0.1 | **0.3 ± 0.1 ***** | **0.2 ± 0.03 ***** |
| ELOvl5 | 1.5 ± 0.4 | **1.7 ± 0.3 ***** | **1.6 ± 0.3 ***** |
| ELOvl6 | 0.9 ± 0.4 | 1.1 ± 0.4 | 1.1 ± 0.4 |
| DGAT1 | 1.5 ± 0.5 | **2.0 ± 0.5 ***** | **2.7 ± 1.1 **** |
| DGAT2 | 1.2 ± 1.0 | 1.1 ± 0.5 | 1.5 ± 0.6 |
| SOAT1 | 1.5 ± 0.5 | **2.0 ± 0.5 ***** | **2.7 ± 1.1 **** |
| CPT1 | 1.0 ± 0.5 | 0.9 ± 0.3 | 1.3 ± 0.4 |
| CPT2 | 1.7 ± 0.6 | **1.7 ± 0.2 ***** | **1.8 ± 0.4 **** |
| CRAT | 1.2 ± 0.5 | 1.1 ± 0.3 | 1.4 ± 0.3 |
| CACT | 1.2 ± 0.5 | 1.1 ± 0.3 | 1.4 ± 0.3 |
| Acox1 | 1.2 ± 0.2 | 1.4 ± 0.3 | 1.6 ± 0.3 |
| Acox2 | **2.4 ± 1.6 ***** | **2.7 ± 2.6 ***** | **2.6 ± 2.0 ***** |

qPCR values normalized against 18S and compared relative to the expression levels in wild type islets. Unpaired student t-test, two tailed, mean ± SD, n = 3, Differences were considered significant when p < 0.01 (** p < 0.01 and *** p < 0.001) as compared to wild type control. ND= not detected
