# Supplementary material for: Susceptibility of Pancreatic Beta Cells to Fatty Acids Is Regulated by LXR/PPARα-Dependent Stearoyl-Coenzyme A Desaturase
Source: PLoS One. 2009 Sep 29;4(9):e7266. doi: 10.1371/journal.pone.0007266 (PMC2746288; doi:10.1371/journal.pone.0007266)
Supplement: Table S3 — mRNA expression levels in beta cells from Zucker obese rats relative to lean controls. qPCR values normalized against β-actin and expressed relative to the expression levels in beta cells from Zucker lean controls. Unpaired student t-test, two tailed, mean±SD, n = 3, Differences were considered significant with ** p<0.01 and *** p<0.001. (0.05 MB DOC) [file pone.0007266.s004.doc]

Table S3: mRNA expression levels in beta cells from Zucker obese rats relative to lean controls.

| **Gene** | **Zucker obese rat** |
| --- | --- |
| LXRα | **1.8 ± 0.3**** |
| LXRβ | 0.9 ± 0.4 |
| PPARα | **0.5 ± 0.1***** |
| FXRα | **0.5 ± 0.1***** |
| SREBP1c | 1.0 ± 0.3 |
| SCD1 | **2.7 ± 0.7**** |
| SCD2 | **2.1 ± 0.4**** |
| ELOvl5 | 1.4 ± 0.5 |
| ELOvl6 | 1.1 ± 0.3 |
| DGAT1 | 1.5 ± 0.4 |
| DGAT2 | 1.1 ± 0.1 |
| SOAT1 | 1.2 ± 0.2 |
| CPT1 | 1.0 ± 0.5 |
| Acad l | 1.0 ± 0.2 |
| Acad m | **0.6 ± 0.1***** |
| Acaa2 | 1.2 ± 0.5 |
| Acaa1 | 1.1 ± 0.1 |
| Acox1 | 0.9 ± 0.2 |
| Acox3 | 1.0 ± 0.2 |

qPCR values normalized against β-actin and expressed relative to the expression levels in beta cells from Zucker lean controls. Unpaired student t-test, two tailed, mean ± SD, n = 3, Differences were considered significant when p < 0.01 (** p < 0.01 and *** p < 0.001) as compared to lean controls.
